# The prolyl 3-hydroxylases P3H2 and P3H3 are novel targets for epigenetic silencing in breast cancer

**DOI:** 10.1038/sj.bjc.6605042

**Published:** 2009-05-12

**Authors:** R Shah, P Smith, C Purdie, P Quinlan, L Baker, P Aman, A M Thompson, T Crook

**Affiliations:** 1 The Breakthrough Toby Robins Breast Cancer Research Centre, Chester Beatty Laboratories, Institute of Cancer Research, Mary-Jean Mitchell Green Building, 237 Fulham Road, London SW3 6JB, UK; 2 Department of Pathology, Ninewells Hospital and Medical School, Dundee DD1 9SY, UK; 3 Department of Surgery and Molecular Oncology, Ninewells Hospital and Medical School, Dundee DD1 9SY, UK; 4 Department of Pathology, Lundberg Laboratory for Cancer Research, Gothenburg University, Gothenburg S-413 45, Sweden

**Keywords:** breast cancer, epigenetics, prolyl hydroxylase

## Abstract

Expression of P3H2 (Leprel1) and P3H3 (Leprel2) but not P3H1 (Leprecan) is down-regulated in breast cancer by aberrant CpG methylation in the 5′ regulatory sequences of each gene. Methylation of P3H2 appears specific to breast cancer as no methylation was detected in a range of cell lines from other epithelial cancers or from primary brain tumours or malignant melanoma. Methylation in P3H2, but not P3H3, was strongly associated with oestrogen-receptor-positive breast cancers, whereas methylation in P3H3 was associated with higher tumour grade and Nottingham Prognostic Index. Ectopic expression of P3H2 and P3H3 in cell lines with silencing of the endogenous gene results in suppression of colony growth. This is the first demonstration of epigenetic inactivation of prolyl hydroxylases in human cancer, implying that this gene family represents a novel class of tumour suppressors. The restriction of silencing in P3H2 to breast carcinomas, and its association with oestrogen-receptor-positive cases, suggests that P3H2 may be a breast-cancer-specific tumour suppressor.

Epigenetics describes heritable changes in gene expression that occur in the absence of changes in DNA sequence (Herman and Baylin, 2003). The best characterised epigenetic alteration in cancer is hypermethylation of CpG rich regions, usually found in the promoter region of a gene (Jones and Baylin, 2002). Hypermethylation, along with other epigenetic events often associated with gene silencing, is crucial in the development of cancer (Baylin, 2005). The detection of methylation-associated gene inactivation is today widely used to identify candidate tumour suppressor genes (Baylin and Ohm, 2006).

The prolyl 3-hydroxylases (P3H), P3H2 and P3H3, were originally termed Leprel1 and Leprel2 due to their ‘Leprecan-like’ amino-acid sequence identity to Leprecan, now termed P3H1 (Wassenhove-McCarthy and McCarthy, 1999; Jarnum *et al*, 2004; Vranka *et al*, 2004). Along with the collagen prolyl 4-hydroxylases (c-P4H) and lysyl hydroxylases (LH), the P3H belong to the 2-oxoglutarate dioxygenases (Vranka *et al*, 2004).

The prolyl 4-hydroxylases (P4H) have been extensively studied and are known to reside in either the endoplasmic reticulum (ER) or cytoplasm, where their function is to hydroxylate proline residues in the X-Pro-Gly sequence in collagens (Kivirikko *et al*, 1989) or to hydroxylate the 564 proline residue in the *α*-subunit of the hypoxia-inducible factor (HIF; Bruick and McKnight, 2001; Epstein *et al*, 2001; Ivan *et al*, 2001). The c-P4H enzymes have a key function in the biosynthesis of collagen allowing appropriate folding of the procollagen chains to form a triple helical structure (Myllyharju, 2003). Furthermore, a decrease in oxygen tension has been found to result in an up-regulation of P4H genes, *P4HA1* and *P4HA2*, as they have been found to be transcriptionally activated by HIF. Although the prolyl hydroxylase reaction does require O_2_, it is thought that the over-production of P4HA1 and P4HA2 in hypoxic conditions compensates for this (Hofbauer *et al*, 2003; Fähling *et al*, 2006).

In comparison to the P4H proteins, the function of the P3H proteins is less well defined. However, it is known that P3H-modified residues are more abundant in basement membrane collagens. Prolyl 3-hydroxylation typically occurs in the Gly-3Hyp-4Hyp sequence (Gryder *et al*, 1975; Vranka *et al*, 2004; Myllyharju, 2005). P3H1 belongs to a family comprising two further genes, all three proteins sharing conserved catalytic residues of the 2-oxoglutarate and iron-dependent dioxygenases with the c-P4Hs and LHs (Vranka *et al*, 2004). P3H2 was initially identified as a protein mainly localised to the endoplasmic reticulum and Golgi (Jarnum *et al*, 2004), but more recently has been demonstrated in tissues rich in basement membranes, and participates in the hydroxylation of collagen IV (Tiainen *et al*, 2008). It has previously been hypothesised that prolyl 3-hydroxylation occurs after prolyl 4-hydroxylation, thus once the triple helix is formed, the 3-hydroxyproline results in destabilisation (Jenkins *et al*, 2003; Mizuno *et al*, 2004). There are no published reports on the function of P3H3.

This study examined the epigenetic regulation of *P3H1*, *P3H2* and *P3H3* expression in breast cancer cell lines and in a panel of breast carcinomas. We show that loss of *P3H2* and *P3H3* expression results from epigenetic silencing, and is associated with aberrant hypermethylation in the CpG islands around exon 1 of both *P3H2* and *P3H3*.

## Materials and methods

### Cell culture

The following breast carcinoma cell lines were used in this study and routinely maintained in Dulbecco's modified Eagle's media (Invitrogen, Paisley, UK), supplemented with L-glutamine (5 mM) and 10% heat-inactivated fetal bovine serum (Invitrogen) in 5% CO_2_: MDA MB 231, MDA MB 361, MDA MB 436, MDA MB 468, MDA MB 453, MCF7, GI101, T47D, NCI, BT474, ZR75, SKBR3 and CAL51. Primary human mammary epithelial cells (HMEC) were cultured using the mammary Epithelial Growth Media bullet kit (Cambrex Corporation, East Rutherford, NJ, USA).

### Expression analysis

Total RNA was extracted using the RNeasy kit (Qiagen Ltd., West Sussex, UK). RNA (500 ng) was used for cDNA synthesis (ImProm-II Reverse Transcription System; Promega, Southampton, UK). Expression of *P3H1*, *P3H2* and *P3H3* was analysed by RT–PCR and normalised to glyceraldehyde-3-phosphate dehydrogenase (GAPDH). Primers were designed using Primer3 software (Totowa, NJ, USA). The primer sequences for RT–PCR were:

*P3H1*: 5′-CTGCAGCACACACCTTCTTC-3′ (forward); 5′-ACAGCTTCCTGTGGCTGTTC-3′ (reverse), product size, 183 bp;

*P3H2*: 5′-TGATGACTTTGAAGGAGGAGAA-3′ (forward); 5′-AGAGCCACAGCACACCTCTT-3′ (reverse), product size, 165bp;

*P3H3*: 5′-GACTGCCTGACCCAGTGC-3′ (forward); 5′-CTGCCAGATCCAGCTTCTTC-3′ (reverse), product size, 153bp;

*GAPDH*: 5′-TGAAGGTCGGAGTCAACGGATTT-3′ (forward); 5′-GCCATGGAATTTGCCATGGGTGG-3′ (reverse), product size, 143 bp.

PCR was performed in a 20 *μ*l volume using 1.8 × ReddyMix PCR Master Mix (Abgene, Epsom, UK). Reaction products were resolved on a 2% agarose gel stained with ethidium bromide, and visualised under UV.

For western blotting, cells were lysed with RIPA lysis buffer. Protein lysate (40 *μ*g) was resolved on 8% SDS–PAGE gel and proteins were transferred onto nitrocellulose membrane that were incubated for 1 h with primary antibodies.

Rabbit antibody against P3H2 was described previously (Jarnum *et al*, 2004) and was used at a dilution of 1 : 1000. Polyclonal rabbit antibodies against P3H3 were raised against the peptide CHQRVQDKTGRAPRVREEL (Biogenes, Berlin, Germany) and used at a dilution of 1 : 1000. The secondary antibody was affinity-purified HRP-conjugated goat anti-rabbit and used at a dilution of 1 : 2000 (Dako, Cambridgeshire, UK). Anti-PCNA (1 : 10 000) was used as a loading control.

### Bisulphite modification

Genomic DNA (gDNA) was extracted from cell pellets using the DNeasy kit (Qiagen Ltd.). Genomic DNA (500 ng) was used for bisulphite modification with the Zymo EZ DNA Methylation kit (Genetix, Hampshire, UK), and eluted in 200 *μ*l dH_2_O. Included in each bisulphite modification were unmethylated human DNA, and CpGenome Universal Methylated DNA (Chemicon International, Temecula, CA, USA), which were used as negative and positive controls, respectively.

### Methylation analysis of the *P3H2* and *P3H3* CpG islands

Methylation was analysed by methylation-specific PCR (MSP) and bisulphite sequencing. Primers were designed using MethPrimer software (http://www.urogene.org/methprimer/).

Methylation-specific PCR primers for *P3H1* were: 5′-GTTTTTTAAGTCGAGGTCGAGTTC-3′ (methylated forward); 5′-ACTAAATACGACAACGCAAACG-3′ (methylated reverse), product size, 180 bp; 5′-TTTTAAGTTGAGGTTGAGTTTGA-3′ (unmethylated forward); 5′-CACTAAATACAACAACACAAACAAA-3′ (unmethylated reverse), product size, 172 bp. Methylation-specific PCR primers for *P3H2* were: 5′-AGAGGGTTTCGGGGTATTTC-3′ (methylated forward 1); 5′-TAAAAACGACTAACCAAACACGAC-3′ (methylated reverse 1), product size, 158 bp; 5′-GAGAGGGTTTTGGGGTATTTT-3′ (unmethylated forward 1); 5′-CTTTAAAAACAACTAACCAAACACAAC-3′ (unmethylated reverse 1), product size, 162 bp. 5′-TTTTTCGTTTTTTGTTGGGGC-3′ (methylated forward 2); 5′-CGAAACGCTAAATCTCACAACTACGAT-3′ (methylated reverse 2), product size, 60 bp. 5′-TTTTGTTTTTTGTTGGGGTGG-3′ (unmethylated forward 2); 5′-CCCCAAAACACTAAATCTCACAACTACA-3′ (unmethylated reverse 2), product size, 61 bp. Methylation-specific PCR primers for *P3H3* were: 5′-GAGGTAAGGTTGGGGTTTTTC-3′ (methylated forward); 5′-CAACCACGTAAACAACTACTACGAT-3′ (methylated reverse), product size, 97 bp; 5′-AGGTAAGGTTGGGGTTTTTTG-3′ (unmethylated forward); 5′-CCCAACCACATAAACAACTACTACA-3′ (unmethylated reverse), product size, 98 bp.

Methylation-specific PCR was performed in a 20 *μ*l volume using Thermo-Start PCR Master Mix (Abgene). The standard thermal cycling conditions were an initial ‘hotstart’ of 8 cycles followed by a further 30 cycles, with a final extension. PCR products were resolved on a 2% agarose gel stained with ethidium bromide (Promega), and visualised under UV.

### Bisulphite sequencing

Bisulphite-modified gDNA was used as the template in the PCR reaction. Primers were designed using MethPrimer software. Primers for *P3H2* were designed spanning the entire predicted CpG island, with a further 200 bp at 5′ and 3′ ends.

Primer sequence for P3H2 were:

5′-ATTTGTATAATTAGAAGGGAGTTTA-3′ (forward); 5′-AACAACAAAAAAAACTCAAAAAAAC-3′ (reverse), product size, 937 bp.

For bisulphite sequence analysis of the *P3H3* CpG island, three sets of primers were designed spanning the CpG island, with a further 200 bp at 5′ and 3′ ends.

Primer sequences for *P3H3* were:

5′-TTGTTGTTATTGTTGTTGTTGTTTTT-3′ (forward 1); 5′-CCCCACCTAATAATAAACCCTCTAC-3′ (reverse 1), product size, 482 bp.

5′-ATTTGTAGAGGGTTTATTAGGTGG-3′ (forward 2); 5′-AACCCTAAACTAAAATAAATACAACC-3′ (reverse 2), product size, 585 bp.

5′-GAGGTAAGGTTGGGGTTTTT-3′ (forward 3); 5′-CTCAATTTAAAAAACCAAATAAAAATAATA-3′ (reverse 3), product size, 230 bp.

Reactions were performed in a 50 *μ*l volume using Thermo-Start PCR Master Mix (Abgene). PCR products were resolved on a 1% agarose gel, with the product of the correct molecular weight excised from the gel, purified using a Gel Extraction kit (Qiagen Ltd.), ligated into the pCR2.1 TA vector (Invitrogen) and transformed into One Shot Top10 Chemically Competent *E. coli* (Invitrogen). Typically, eight colonies were picked per cell line, and sequenced with the reverse primer, using the BigDye Terminator v1.1 Cycle Sequencing kit (Applied Biosystems, Foster City, CA, USA).

### Clinical tissue

Genomic DNA was extracted from 184 primary, previously untreated breast cancers, using the M48 Qiagen DNA extraction robot, following Tayside Tissue Bank Local Research Ethics Committee approval. Cancers were subject to histopathological review before use for DNA extraction to ensure adequate representation of neoplastic cells. Expression of the oestrogen receptor, progesterone receptor and HER2 (using antibody CB11, supplemented by FISH for 2-positive cancers to confirm amplification), was measured as part of routine clinical care. Clinical and pathological data included tumour grade, tumour type, pathology node status, and relapse-free and overall survival.

### Colony formation assay

Plasmids for ectopic expression of *P3H2* and *P3H3* were as follows: pEGFP-N1-*P3H2* was as previously described (Jarnum *et al*, 2004). A full-length P3H3 cDNA in pCMV6-XL6 vector was purchased from Origene (Rockville, MD, USA) and the insert was subcloned into pcDNA3.1 as a *Not*1 fragment. Correct orientation was determined by sequencing multiple plasmid clones. To assess the effect on cell proliferation of ectopic expression of *P3H2* and *P3H3*, cell lines were transfected with 4 *μ*g of the above expression clones or empty vector alone, using Lipofectamine 2000 (Invitrogen). Media were changed 24 h after transfection, and transfected cells were selected in G418 (800 *μ*g ml^−1^). After 16-day growth, surviving colonies were fixed in 4% paraformaldehyde, washed with phosphate-buffered saline, and dH_2_O, dried, then stained with liquid crystal violet (Sigma-Aldrich, Dorset, UK) and counted. Experiments were carried out in triplicate.

### Statistical analyses

Assessment between two categorical variables was carried out using *χ*^2^- or Fisher's exact test. Analysis of the cumulative survival was carried out by the Kaplan–Meier method and differences between the groups were tested with the log-rank test. All reported *P*-values were two sided and considered statistically significant if *P*<0.05. Tests were performed using GraphPad Prism version 5.0 software (GraphPad Software Inc, San Diego, CA, USA).

## Results

### Transcriptional down-regulation of *P3H2* and *P3H3* in breast cancer cell lines

**Figure 1:**
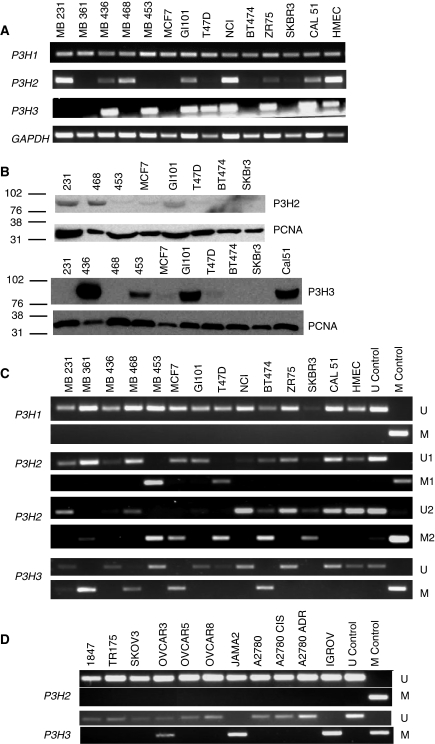
Epigenetic regulation of expression of *P3H2* and *P3H3* in breast carcinoma cell lines. (**A**) RT–PCR analysis of *P3H1*, *P3H2* and *P3H3* expression in the indicated breast carcinoma cell lines and normal breast epithelium (HMEC). The control gene *GAPDH* is also shown. (**B**) Western blot analysis of expression of P3H2 and P3H3 in breast carcinoma cell lines. Western blot analysis of the indicated breast carcinoma cell lines was performed as described in Materials and methods. The control gene PCNA is also shown. Approximate position of molecular weight markers is indicated. (**C**) Methylation in the CpG islands of *P3H2* and *P3H3* correlates with down-regulation of expression. As shown, the CpG island of *P3H1* was uniformly unmethylated in each cell line and in normal breast epithelium (HMEC), consistent with expression analysis (**A**). The figure shows MSP analysis of the *P3H2* CpG island using two primer pairs. Pair 1 (upper panel of *P3H2*) detects methylation in the MDA MB 453 and T47D cell lines, both of which lacked detectable expression. Primer pair 2 (lower panel of *P3H2*) that is located further 3′ in the CpG island detected methylation in MDA MB 361, MDA MB 453, MCF7, T47D, BT474 and SKBR3 cell lines, which correlates closely with expression analysis. Methylation-specific PCR analysis of the *P3H3* CpG island detects methylation in MDA MB 231, MDA MB 361, MDA MB 468, MCF7, BT474 and SKBR3, showing a clear correlation between methylation and down-regulation of mRNA. (**D**) Methylation-specific PCR analysis of *P3H2* and *P3H3* genes in ovarian carcinoma cell lines. The *P3H3* CpG island is clearly methylated in OVCAR3, JAMA2 and IGROV cell lines. In contrast, there is no evidence of methylation in the *P3H2* CpG island in any of the cell lines analysed.

Using RT–PCR, we analysed the expression of the *P3H1*, *P3H2* and *P3H3* genes in breast carcinoma cell lines (Figure 1A). All three genes were expressed in HMEC. *P3H1* was expressed in all 13 carcinoma cell lines in our panel, but there was no detectable expression of *P3H2* mRNA in MDA MB 361, MDA MB 453, MCF7 and T47D cell lines, with only low levels of expression in BT474 and SKBR3. In the case of *P3H3*, expression was undetectable in the MDA MB 231, MDA MB 361, MDA MB 468, MCF7, BT474 and SKBR3 cell lines (Figure 1A). Next, we analysed protein levels of P3H2 and P3H3. We used a previously described antibody to P3H2 and generated a new polyclonal antibody to P3H3 and performed western analysis of the breast carcinoma cell line panel. In general, protein levels for both P3H2 and P3H3 paralleled mRNA expression (Figure 1A and 1B). Interestingly, however, P3H3 protein was barely detectable in T47D cells despite readily detectable expression of *P3H3* mRNA, and the level of P3H3 protein was also reduced in MDA MB 453 relative to MDA MB 436, GI101 and Cal51 despite comparable expression of *P3H3* mRNA. This may reflect other regulatory mechanisms operating at the level of mRNA translation or protein stability.

### Aberrant methylation of *P3H2* and *P3H3* in breast cancer cell lines

We identified CpG islands in the 5′ sequences of *P3H1*, *P3H2* and *P3H3* genes (http://genome.ucsc.edu). To address whether promoter methylation was the cause of loss of gene expression, we performed MSP analysis of the CpG islands of *P3H1*, *P3H2* and *P3H3* in each of the breast cancer cell lines. The CpG island of *P3H1* was uniformly unmethylated in each cell line consistent with expression analysis (Figure 1C). In the case of *P3H2* methylation, initial analysis used primers located in the centre of the CpG island and these detected methylation in the MDA MB 453 and T47D cell lines, both of which lacked detectable expression of *P3H2*. However, analysis with this primer set did not detect methylation in some cell lines that lack expression of P3H2. We therefore designed a second primer set located further 3′ in the CpG island. Analysis of the cell line panel with this primer set detected methylation additionally in MDA MB 361, MCF7, BT474 and SKBR3, as well as MDA MB 453 and T47D (Figure 1C), establishing a good correlation with down-regulation of mRNA. Methylation-specific PCR analysis in the *P3H3* CpG island with a single primer set detected methylation in cell lines MDA MB 231, MDA MB 361, MDA MB 468, MCF7, BT474 and SKBR3 (Figure 1C), confirming a clear correlation between methylation as detected by MSP and down-regulation of mRNA.

**Figure 2:**
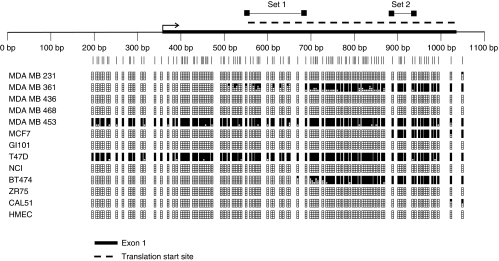
Schematic representation of bisulphite sequence analysis of the *P3H2* CpG island in breast carcinoma cell lines and normal breast epithelium (HMEC). Bisulphite sequencing was performed as described in Materials and methods. Primer pairs 1 and 2 used for MSP are indicated as set 1 and set 2 respectively. The thick black line on the scale indicates the position of exon 1 relative to the CpG island. The position of the part of the P3H2 open-reading frame within the CpG island is indicated by the broken line above the scale. Vertical lines below the scale represent individual CpG dinucleotides within the CpG island. The density of methylation for each cell line is represented by a quartile of blocks corresponding to each CpG. Black shading represents up to 25% methylation. Open blocks indicate no methylation. There is dense methylation in the MDA MB 361, MDA MB 453, MCF7, T47D and BT474 cell lines. There is no methylation in HMEC.

**Figure 3:**
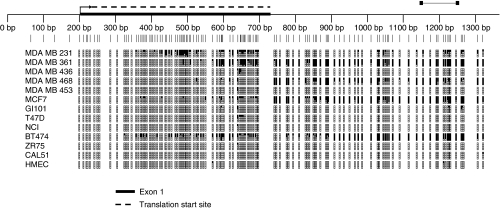
Schematic representation of bisulphite sequence analysis of the *P3H3* CpG island in breast carcinoma cell lines and normal breast epithelium (HMEC). Bisulphite sequencing was performed as described in Materials and methods. Primers used for MSP are indicated above the scale. The thick black line on the scale indicates the position of exon 1 relative to the CpG island. The position of the part of the *P3H3* open-reading frame within the CpG island is indicated by the broken line above the scale. Vertical lines below the scale represent individual CpG dinucleotides within the CpG island. The density of methylation for each cell line is represented by a quartile of blocks corresponding to each CpG. Black shading represents up to 25% methylation. Open blocks indicate no methylation. There is methylation in MDA MB 231, MDA MB 361, MDA MB 468, MCF7 and BT474. The CpG island is uniformly unmethylated in HMEC.

To characterise methylation in greater detail across the *P3H2* and *P3H3* CpG islands, we mapped each island, using bisulphite sequencing, in a panel of cell lines previously analysed by MSP (Figures 2 and 3). These studies closely paralleled the MSP analysis. For example, in the *P3H2* CpG island, the region of the CpG island sampled by MSP primer pair 1 was methylated only in the MDA MB 453 and T47D cell lines. In contrast, the region of the CpG island sampled by MSP primer pair 2 contained methylation in all cell lines lacking expression of P3H2. Consistent with methylation-dependent transcriptional silencing, some of the cell lines expressing P3H2 mRNA show an extremely low frequency of CpG methylation (Figure 2). In the case of *P3H3*, methylation was observed across the entire CpG island consistent with MSP analysis (Figure 3). As with *P3H2*, there was an extremely low level of methylation in some of the cell lines that express P3H3 mRNA (Figure 3). The entire CpG islands of both *P3H2* and *P3H3* were unmethylated in normal mammary epithelium.

### Methylation of *P3H2* is specific for breast carcinomas

The observation of methylation-dependent transcriptional silencing in *P3H2* and *P3H3* prompted us to examine expression and methylation of the P3H genes in cell lines from other common solid tumour types. In ovarian, head and neck, vulval, melanoma, glioblastoma and renal carcinoma cell lines, *P3H3* was clearly methylated and, as in breast cancer, this correlated with down-regulation of the mRNA (Figure 1D; data not shown). In contrast, we found no evidence for methylation of *P3H2* in analysis of cell lines from multiple other tumour types, including ovarian and renal adenocarcinomas, squamous carcinomas of the vulva and head and neck, malignant melanoma and glioma (Figure 1D; data not shown). These results imply that whereas *P3H3* is widely methylated in human cancers, methylation in *P3H2* is restricted to breast cancer, at least within the tumour types we have analysed in the present study.

### *P3H2* and *P3H3* genes are methylated in primary breast carcinomas

**Figure 4:**
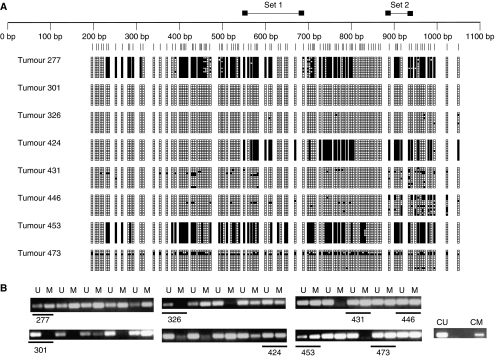
The *P3H2* and *P3H3* CpG islands are methylated in primary breast carcinomas. (**A**) Bisulphite sequence analysis of *P3H2* CpG island in eight randomly selected primary breast carcinomas. The figure shows a schematic representation of the *P3H2* CpG island as described in legend for Figure 2. Vertical lines below the scale represent individual CpG dinucleotides within the CpG island. The density of methylation for each cell line is represented by a quartile of blocks corresponding to each CpG. Black shading represents up to 25% methylation. Open blocks indicate no methylation. (**B**) Methylation-specific PCR analysis of *P3H2* in primary breast carcinomas. It is shown with the number of each carcinoma analysed by both MSP and bisulphite sequencing indicated (underlined in the MSP gel). Unmethylated (CU) and methylated (CM) control DNAs, modified in parallel with primary cancer DNA samples in each case, are also shown. Cases 277, 424, 431, 446, 453 and 473 were identified as positive for methylation by MSP with primer set 2 as shown, whereas cases 301 and 326 were negative confirming the sensitivity and specificity of this primer set for methylation detection.

Next we tested whether the CpG islands of *P3H2* and *P3H3* are methylated in a series of 184 primary breast carcinomas. From studies in cell lines described above, MSP analysis with primer set 2 of *P3H2* was most strongly associated with down-regulated expression of the mRNA. To further confirm the utility of this primer set for methylation detection, we performed preliminary bisulphite sequencing on eight cancers designated as either methylated or unmethylated by MSP. These initial studies fully confirmed that primer pair 2 accurately assessed methylation status and so this primer pair was used for all subsequent analyses of the full set of cases (Figure 4). The frequency of methylation for *P3H2* and *P3H3* found in the 184 analysed breast samples was 42 and 26% respectively. Several associations were observed between methylation status and clinicopathological parameters (Table 1). First, methylation in the *P3H2* CpG island was positively associated with a positive oestrogen receptor status (*P*=0.0053), whereas methylation in the *P3H3* CpG island showed no such association (*P*=0.71) (Table 1). The observation prompted us to determine whether there was a similar association in breast cancer cell lines. Other than MDA MB 453, all cell lines methylated in the P3H2 CpG island (MDA MB 361, MDA MB 453, MCF7, T47D, BT474 and SKBR3 express the oestrogen receptor). Methylation of the *P3H3* CpG island was positively associated with increasing tumour grade (*P*=0.02) and with higher Nottingham Prognostic Index (*P*=0.02). However, we did not find evidence that methylation in either gene was associated with clinical outcome (Table 1).

### Ectopic expression of *P3H2* and *P3H3* suppresses colony-forming ability

**Figure 5:**
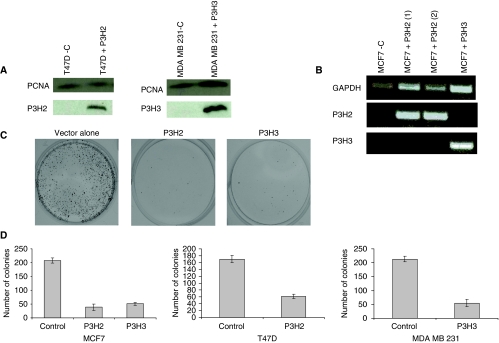
Ectopic expression of *P3H2* and *P3H3* in cells lacking endogenous expression suppresses cell proliferation. Expression plasmids for each gene or empty vector alone were introduced into individual cell lines as shown and transfected cells selected in G418. Surviving colonies were stained and counted after 16 days. (**A**) Expression of transfected plasmids for *P3H2* and *P3H3*. T47D cells (which lack endogenous *P3H2*) and MDA MB 231 cells (which lack endogenous *P3H3*) were transfected with either control vector (**C**) or *P3H2* and *P3H3* expression plasmids respectively as indicated. Cell lysates were prepared and subjected to western blot analysis as described in Materials and methods. (**B**) Expression of transfected plasmids for *P3H2* and *P3H3* in MCF7 cells. MCF7 cells, which lack endogenous expression of both *P3H2* and *P3H3*, were transfected with the indicated expression plasmids. Expression of transfected plasmids was analysed by RT–PCR as described in Materials and Methods. (**C**) Representative experiment showing suppression of colony growth by ectopic expression of *P3H2* and *P3H3*. MCF7 cells (which lack endogenous expression of both *P3H2* and *P3H3*) were transfected with expression plasmids for *P3H2* and *P3H3* as indicated. Cells were grown in the presence of G418 for 16 days, and surviving colonies were stained and counted. (**D**) Suppression of colony growth of MCF7, T47D and MDA MB 231 cells by ectopic expression of *P3H2* and *P3H3*. The data shown are mean number of colonies (±standard error of mean) from three independent plates.

Genes found to be hypermethylated in their promoter region are often considered to be candidate tumour suppressor genes. One property that such genes may possess is the ability to suppress proliferation when ectopically expressed in cells lacking endogenous expression. To determine whether this was the case for P3H2 and P3H3, an expression plasmid for *P3H2* was introduced into MCF7 and T47D cell lines and an expression plasmid for *P3H3* was introduced into MCF7 and MDA MB 231, and we assessed the efficiency of colony formation after 16 days in G418 selection. In each case, expression of the transfected cDNA efficiently suppressed colony growth (Figure 5). Using RT–PCR and western blotting we confirmed that the transfected sequences were expressed (Figure 5). Ability to suppress proliferation demonstrated in these assays is consistent with a potential tumour suppressor function for P3H2 and P3H3.

## Discussion

The 2-oxoglutarate dioxygenases are a family of proteins required for modifications of collagen that are essential for its synthesis, folding and assembly. The collagen P3H are members of the 2-oxoglutarate dioxygenase family, which catalyse the post-translational formation of 3-hydroxyproline in Gly-3Hyp-4Hyp sequences in collagens, especially type IV and V collagens. The possible involvement of the *P3H* genes in human tumourigenesis was explored because ectopic expression of P3H1 was reported to cause growth arrest in fibroblasts (Kaul *et al*, 2000). There are three *P3H* proteins encoded in the human genome, *P3H1*, *P3H2* and *P3H3*. Here, we show that both *P3H2* and *P3H3*, but not *P3H1*, are frequent targets for epigenetic inactivation in human breast cancer. To the best of our knowledge, this is the first to report of an epigenetic inactivation of any prolyl hydroxylase gene in human neoplasia.

Breast carcinoma cell lines were screened by RT–PCR and western blotting for expression of the three P3H genes. Strikingly, whereas P3H1 was present in all cell lines, expression of both P3H2 and P3H3 was undetectable in several lines at both mRNA and protein levels. Bisulphite sequencing and MSP of the CpG islands located in the 5′ sequences of each gene revealed a clear correlation between down-regulated expression and aberrant methylation for both genes. This implies that methylation-dependent transcriptional silencing is the mechanistic basis for the loss of mRNA expression, as is the case for a number of tumour suppressor genes in breast cancer, including *p16*^*INK4a*^, *Rassf1a* and *E-cadherin* among others. Previous expression profiling studies of human breast cancer show that *P3H2* mRNA (Radvanyi *et al*, 2005; Richardson *et al*, 2006) and *P3H3* mRNA (van't Veer *et al*, 2002) are down-regulated in breast cancer, consistent with our results.

Taken together, our results suggest that P3H2 and P3H3 are candidate tumour suppressors in breast cancer, raising the question of which function(s) are selected against during tumourigenesis. The collagen prolyl hydroxylases are localised to the endoplasmic reticulum and their activity is required for proper collagen synthesis and assembly. Studies of inherited disorders of collagen biosynthesis suggest that loss of function in P3H proteins results in dysfunctional collagen; mutations in P3H1 are associated with oesteogenesis imperfecta type VIII (Cabral *et al*, 2007) and loss of function mutations in both P3H1- and P3H-related protein CRTAP have been described in oesteogenesis imperfecta types II and III (Baldridge *et al*, 2008). Evidence implicating collagen abnormalities in human tumours is afforded by studies showing methylation-dependent silencing of collagen-encoding genes in various tumour types (Sengupta *et al*, 2003; Ikeda *et al*, 2006). Type IV collagen, a major substrate for the P3H proteins, is an important component of the basement membrane, and impaired expression of type IV collagen has been reported to be an early event in acquisition of an invasive phenotype in some epithelial cancers (Ikeda *et al*, 2006). In this respect, it will clearly be of interest to determine whether loss of expression of P3H2 and/or P3H3 affects the properties of the basement membrane in breast cancer cells and thereby influences cancer-associated phenotypes such as invasiveness and metastasis. In addition to the possible effects of loss of P3H2 and P3H3 on collagen, our data demonstrate that both P3H2 and P3H3 have a direct anti-proliferative effect in breast cancer, suggesting additional tumour suppressor properties for each gene. Specifically, ectopic expression of both P3H2 and P3H3 in cells lacking endogenous expression due to epigenetic silencing resulted in a decrease in colony formation. Inhibition of colony formation in such assays has been demonstrated previously with known tumour suppressors such as p53 (Crook *et al*, 1994). Clearly, understanding the mechanism(s) by which the P3H genes negatively regulate proliferation will require additional studies.

A striking feature of the data is the restriction of methylation in *P3H2* to breast cancer, with no detectable methylation in carcinoma cell lines in the other tumour types examined. Such tight specificity of methylation in one gene for a single tumour type is unusual and raises the potential to use detection of methylated DNA either in tissue or body fluids as a cancer biomarker. Verification that *P3H2* is methylated only in breast cancer would make it an attractive candidate gene with potential utility in diagnosis and screening in breast cancer. The specificity of down-regulation of *P3H2* mRNA in breast cancer may reflect the association with oestrogen-receptor-positive primary breast cancers, an association also noted in breast carcinoma cell lines. Selective methylation of the *P3H2* CpG island in oestrogen-receptor-positive breast cancers is consistent with multiple array-based expression profiling studies (Miller *et al*, 2005; Minn *et al*, 2005; Wang *et al*, 2005; Hess *et al*, 2006) and in array analysis comparing oestrogen-receptor-positive and -negative breast cancer cell lines (Neve *et al*, 2006). However, it remains to be determined whether *P3H2* is an oestrogen-inducible gene and what the mechanistic basis is for the selective methylation of *P3H2* in oestrogen-receptor-positive cases. One possibility is that *P3H2* is an oestrogen-inducible negative regulator of proliferation. This hypothesis is supported by the demonstration that ectopic expression of P3H2 in cell lines lacking endogenous expression suppresses colony survival and growth. A second interesting association was that methylation in *P3H3* was associated with higher histopathological grade, consistent with a number of expression profiling studies (van't Veer *et al*, 2002; Ivshina *et al*, 2004; Zhao *et al*, 2004; Farmer *et al*, 2005; Miller *et al*, 2005; Ginestier *et al*, 2006; Hess *et al*, 2006) and with higher Nottingham Prognostic Index. From the relatively small number of cases of primary breast cancer analysed (*n*=184), we did not observe a significant association between *P3H2* or *P3H3* methylation and clinical outcome. However, mRNA analysis implies that down-regulation of P3H2 is associated with less favourable prognosis in some breast cancer series (van de Vijver *et al*, 2002; Pawitan *et al*, 2006; Desmedt *et al*, 2007) and recurrence after tamoxifen (Ma *et al*, 2004). It will clearly be of interest to determine in large study populations whether analysis of *P3H2* methylation has prognostic utility in breast cancer.

This is the first demonstration of epigenetic inactivation of prolyl hydroxylases in human cancer. The prolyl 3-hydroxylases *P3H2* and *P3H3* are, therefore, novel candidate tumour suppressor genes in breast cancer.

## Figures and Tables

**Table 1:** Relationship between (A) P3H2 methylation and clinical parameters and (B) P3H3 methylation and clinical parameters

	***n***	**U**	**M**	***p***	**OS**	**DFS**
(A)						
Total	184	107	77	—	0.9136	0.8587
						
*ER status*						
ER^+^	130	67	63	0.0053	0.3681	0.4207
ER^−^	54	40	14		0.9874	0.5983
						
*PgR status*						
PgR^+^	77	40	37	0.1736	0.0656	0.0677
PgR^−^	107	67	40		0.5742	0.5379
						
*Menopausal*						
Pre	35	20	15	0.1021	0.8266	0.7161
Peri	6	6	0		NA	NA
Post	141	79	62		0.9941	0.9951
Unknown	2	2	0		NA	NA
						
*Tumour grade*						
1	32	20	12	0.7497	0.9147	0.6178
2	67	37	30		0.3671	0.8012
3	75	45	30		0.2621	0.8066
Unknown	10	5	5			
						
*NPI*						
1 (good)	42	26	16	0.7725	0.9865	0.404
2 (moderate)	79	46	33		0.8471	0.6637
3 (poor)	46	25	21		0.6778	0.1847
Unknown	17	10	7			
						
*p53 status*						
WT	130	73	57	0.4167	0.4665	0.5849
Mutant	54	34	20		0.4091	0.5051
						
*Cell lines*						
ER^+^		1	6	0.029		
ER^−^		6	1			
						
(B)						
Total	184	136	48	—	0.7504	0.3621
						
*ER status*						
ER^+^	130	97	33	0.717	0.6233	0.7558
ER^−^	54	39	15		0.2504	0.1384
						
*PgR status*						
PgR^+^	77	61	16	0.1776	0.8762	0.9168
PgR^−^	107	75	32		0.3545	0.1295
						
*Menopausal*						
Pre	35	26	9	0.8508	0.6927	0.5699
Peri	6	5	1		0.6547	0.6547
Post	141	103	38		0.9136	0.5579
Unknown	2	2	0		NA	NA
						
*Tumour grade*						
1	32	29	3	0.02	0.372	0.7803
2	67	48	19		0.9137	0.4962
3	75	54	21		0.4772	0.5206
Unknown	10	5	5			
						
*NPI*						
1 (good)	42	38	4	0.02	0.9638	0.9422
2 (moderate)	79	58	21		0.5954	0.1292
3 (poor)	46	31	15		0.2346	0.446
Unknown	17	8	9			
						
*p53 status*						
WT	130	96	34	1	0.6751	0.99
Mutant	54	40	14		0.4247	0.2493

DFS=disease-free survival; ER=oestrogen receptor; NA=not applicable; NPI=Nottingham Prognostic Index; OS=overall survival; PgR=progesterone receptor; WT=wild type.

## References

[bib1] Baldridge D, Schwarze U, Morello R, Lennington J, Bertin TK, Pace JM, Pepin MG, Weis M, Eyre DR, Walsh J, Lambert D, Green A, Robinson H, Michelson M, Houge G, Lindman C, Martin J, Ward J, Lemyre E, Mitchell JJ, Krakow D, Rimoin DL, Cohn DH, Byers PH, Lee B (2008) CRTAP and LEPRE1 mutations in recessive osteogenesis imperfecta. Hum Mutat 29: 1435–1442 18566967 10.1002/humu.20799 PMC2671575

[bib2] Baylin SB (2005) DNA methylation and gene silencing in cancer. Nat Clin Pract Oncol 2Suppl (1): S4–S11 16341240 10.1038/ncponc0354

[bib3] Baylin SB, Ohm JE (2006) Epigenetic gene silencing in cancer – a mechanism for early oncogenic pathway addiction? Nat Rev Cancer 6: 107–116 16491070 10.1038/nrc1799

[bib4] Bruick RK, McKnight SL (2001) A conserved family of prolyl-4-hydroxylases that modify HIF. Science 294: 1337–1340 11598268 10.1126/science.1066373

[bib5] Cabral WA, Chang W, Barnes AM, Weis M, Scott MA, Leikin S, Makareeva E, Kuznetsova NV, Rosenbaum KN, Tifft CJ, Bulas DI, Kozma C, Smith PA, Eyre DR, Marini JC (2007) Prolyl 3-hydroxylase 1 deficiency causes a recessive metabolic bone disorder resembling lethal/severe osteogenesis imperfecta. Nat Genet 39: 359–365 17277775 10.1038/ng1968 PMC7510175

[bib6] Crook T, Marston NJ, Sara EA, Vousden KH (1994) Transcriptional activation by p53 correlates with suppression of growth but not transformation. Cell 79: 817–827 8001119 10.1016/0092-8674(94)90071-x

[bib7] Desmedt C, Piette F, Loi S, Wang Y, Lallemand F, Haibe-Kains B, Viale G, Delorenzi M, Zhang Y, d'Assignies MS, Bergh J, Lidereau R, Ellis P, Harris AL, Klijn JG, Foekens JA, Cardoso F, Piccart MJ, Buyse M, Sotiriou C (2007) Strong time dependence of the 76-gene prognostic signature for node-negative breast cancer patients in the TRANSBIG multicenter independent validation series. Clin Cancer Res 13: 3207–3214 17545524 10.1158/1078-0432.CCR-06-2765

[bib8] Epstein AC, Gleadle JM, McNeill LA, Hewitson KS, O'Rourke J, Mole DR, Mukherji M, Metzen E, Wilson MI, Dhanda A, Tian YM, Masson N, Hamilton DL, Jaakkola P, Barstead R, Hodgkin J, Maxwell PH, Pugh CW, Schofield CJ, Ratcliffe PJ (2001) *C elegans* EGL-9 and mammalian homologs define a family of dioxygenases that regulate HIF by prolyl hydroxylation. Cell 107: 43–54 11595184 10.1016/s0092-8674(01)00507-4

[bib9] Fähling M, Mrowka R, Steege A, Nebrich G, Perlewitz A, Persson PB, Thiele BJ (2006) Translational control of collagen prolyl 4-hydroxylase-*α*(I) gene expression under hypoxia. J Biol Chem 281: 26089–26101 16837461 10.1074/jbc.M604939200

[bib10] Farmer P, Bonnefoi H, Becette V, Tubiana-Hulin M, Fumoleau P, Larsimont D, Macgrogan G, Bergh J, Cameron D, Goldstein D, Duss S, Nicoulaz AL, Brisken C, Fiche M, Delorenzi M, Iggo R (2005) Identification of molecular apocrine breast tumours by microarray analysis. Oncogene 24: 4660–4671 15897907 10.1038/sj.onc.1208561

[bib11] Ginestier C, Cervera N, Finetti P, Esteyries S, Esterni B, Adélaïde J, Xerri L, Viens P, Jacquemier J, Charafe-Jauffret E, Chaffanet M, Birnbaum D, Bertucci F (2006) Prognosis and gene expression profiling of 20q13-amplified breast cancers. Clin Cancer Res 12: 4533–4544 16899599 10.1158/1078-0432.CCR-05-2339

[bib12] Gryder RM, Lamon M, Adams E (1975) Sequence position of 3-hydroxyproline in basement membrane collagen. Isolation of glycyl-3-hydroxyprolyl-4-hydroxyproline from swine kidney. J Biol Chem 250: 2470–2474 164442

[bib13] Herman JG, Baylin SB (2003) Gene silencing in cancer in association with promoter hypermethylation. N Engl J Med 349: 2042–2054 14627790 10.1056/NEJMra023075

[bib14] Hess KR, Anderson K, Symmans WF, Valero V, Ibrahim N, Mejia JA, Booser D, Theriault RL, Buzdar AU, Dempsey PJ, Rouzier R, Sneige N, Ross JS, Vidaurre T, Gómez HL, Hortobagyi GN, Pusztai L (2006) Pharmacogenomic predictor of sensitivity to preoperative chemotherapy with paclitaxel and fluorouracil, doxorubicin, and cyclophosphamide in breast cancer. J Clin Oncol 24: 4236–4244 16896004 10.1200/JCO.2006.05.6861

[bib15] Hofbauer KH, Gess B, Lohaus C, Meyer HE, Katschinski D, Kurtz A (2003) Oxygen tension regulates the expression of a group of procollagen hydroxylases. Eur J Biochem 270: 4515–4522 14622280 10.1046/j.1432-1033.2003.03846.x

[bib16] Ikeda K, Iyama K, Ishikawa N, Egami H, Nakao M, Sado Y, Ninomiya Y, Baba H (2006) Loss of expression of type IV collagen alpha5 and alpha6 chains in colorectal cancer associated with the hypermethylation of their promoter region. Am J Pathol 168: 856–865 16507901 10.2353/ajpath.2006.050384 PMC1606532

[bib17] Ivan M, Kondo K, Yang H, Kim W, Valiando J, Ohh M, Salic A, Asara JM, Lane WS, Kaelin Jr WG (2001) HIFalpha targeted for VHL-mediated destruction by proline hydroxylation: implications for O2 sensing. Science 292: 464–468 11292862 10.1126/science.1059817

[bib18] Ivshina AV, George J, Senko O, Mow B, Putti TC, Smeds J, Lindahl T, Pawitan Y, Hall P, Nordgren H, Wong JE, Liu ET, Bergh J, Kuznetsov VA, Miller LD (2004) Genetic reclassification of histologic grade delineates new clinical subtypes of breast cancer. Cancer Res 66: 10292–10301 10.1158/0008-5472.CAN-05-4414 17079448

[bib19] Jarnum S, Kjellman C, Darabi A, Nilsson I, Edvardsen K, Aman P (2004) Leprel1, a novel ER and Golgi resident member of the Leprecan family. Biochem Biophys Res Commun 317: 342–351 15063763 10.1016/j.bbrc.2004.03.060

[bib20] Jenkins CL, Bretscher LE, Guzei IA, Raines RT (2003) Effect of 3-hydroxyproline residues on collagen stability. J Am Chem Soc 125: 6422–6427 12785781 10.1021/ja034015j

[bib21] Jones PA, Baylin SB (2002) The fundamental role of epigenetic events in cancer. Nat Rev Genet 3: 415–428 12042769 10.1038/nrg816

[bib22] Kaul SC, Sugihara T, Yoshida A, Nomura H, Wadhwa R (2000) Gros1, a potential growth suppressor on chromosome 1: its identity to basement membrane-associated proteoglycan, leprecan. Oncogene 19: 3576–3583 10951563 10.1038/sj.onc.1203696

[bib23] Kivirikko KI, Myllylä R, Pihlajaniemi T (1989) Protein hydroxylation: prolyl 4-hydroxylase, an enzyme with four co-substrates and a multifunctional subunit. FASEB J 3: 1609–1617 2537773

[bib24] Ma XJ, Wang Z, Ryan PD, Isakoff SJ, Barmettler A, Fuller A, Muir B, Mohapatra G, Salunga R, Tuggle JT, Tran Y, Tran D, Tassin A, Amon P, Wang W, Wang W, Enright E, Stecker K, Estepa-Sabal E, Smith B, Younger J, Balis U, Michaelson J, Bhan A, Habin K, Baer TM, Brugge J, Haber DA, Erlander MG, Sgroi DC (2004) A two-gene expression ratio predicts clinical outcome in breast cancer patients treated with tamoxifen. Cancer Cell 5: 607–616 15193263 10.1016/j.ccr.2004.05.015

[bib25] Miller LD, Smeds J, George J, Vega VB, Vergara L, Ploner A, Pawitan Y, Hall P, Klaar S, Liu ET, Bergh J (2005) An expression signature for p53 status in human breast cancer predicts mutation status, transcriptional effects, and patient survival. Proc Natl Acad Sci 102: 13550–13555 16141321 10.1073/pnas.0506230102 PMC1197273

[bib26] Minn AJ, Gupta GP, Siegel PM, Bos PD, Shu W, Giri DD, Viale A, Olshen AB, Gerald WL, Massagué J (2005) Genes that mediate breast cancer metastasis to lung. Nature 436: 518–524 16049480 10.1038/nature03799 PMC1283098

[bib27] Mizuno K, Hayashi T, Peyton DH, Bachinger HP (2004) The peptides acetyl-(Gly-3(S)Hyp-4(R)Hyp)10-NH2 and acetyl-(Gly-Pro-3(S)Hyp)10-NH2 do not form a collagen triple helix. J Biol Chem 279: 282–287 14576161 10.1074/jbc.M308181200

[bib28] Myllyharju J (2003) Prolyl 4-hydroxylases, the key enzymes of collagen biosynthesis. Matrix Biol 22: 15–24 12714038 10.1016/s0945-053x(03)00006-4

[bib29] Myllyharju J (2005) Intracellular Post-Translational Modifications of Collagens. Springer: Berlin/Heidelberg 2005; 247: 115–147

[bib30] Neve RM, Chin K, Fridlyand J, Yeh J, Baehner FL, Fevr T, Clark L, Bayani N, Coppe JP, Tong F, Speed T, Spellman PT, DeVries S, Lapuk A, Wang NJ, Kuo WL, Stilwell JL, Pinkel D, Albertson DG, Waldman FM, McCormick F, Dickson RB, Johnson MD, Lippman M, Ethier S, Gazdar A, Gray JW (2006) A collection of breast cancer cell lines for the study of functionally distinct cancer subtypes. Cancer Cell 10: 515–527 17157791 10.1016/j.ccr.2006.10.008 PMC2730521

[bib31] Pawitan Y, Bjöhle J, Amler L, Borg AL, Egyhazi S, Hall P, Han X, Holmberg L, Huang F, Klaar S, Liu ET, Miller L, Nordgren H, Ploner A, Sandelin K, Shaw PM, Smeds J, Skoog L, Wedrén S, Bergh J (2006) Gene expression profiling spares early breast cancer patients from adjuvant therapy: derived and validated in two population-based cohorts. Breast Cancer Res 7: R953–R964 10.1186/bcr1325 PMC1410752 16280042

[bib32] Radvanyi L, Singh-Sandhu D, Gallichan S, Lovitt C, Pedyczak A, Mallo G, Gish K, Kwok K, Hanna W, Zubovits J, Armes J, Venter D, Hakimi J, Shortreed J, Donovan M, Parrington M, Dunn P, Oomen R, Tartaglia J, Berinstein NL (2005) The gene associated with trichorhinophalangeal syndrome in humans is overexpressed in breast cancer. Proc Natl Acad Sci USA 102: 11005–11100 16043716 10.1073/pnas.0500904102 PMC1182410

[bib33] Richardson AL, Wang ZC, De Nicolo A, Lu X, Brown M, Miron A, Liao X, Iglehart JD, Livingston DM, Ganesan S (2006) X chromosomal abnormalities in basal-like human breast cancer. Cancer Cell 9: 121–132 16473279 10.1016/j.ccr.2006.01.013

[bib34] Sengupta PK, Smith EM, Kim K, Murnane MJ, Smith BD (2003) DNA hypermethylation near the transcription start site of collagen alpha2(I) gene occurs in both cancer cell lines and primary colorectal cancers. Cancer Res 63: 1789–1797 12702564

[bib35] Tiainen P, Pasanen A, Sormunen R, Myllyharju J (2008) Characterization of recombinant human prolyl 3-hydroxylase isoenzyme 2, an enzyme modifying the basement membrane collagen IV. J Biol Chem 283: 19432–19439 18487197 10.1074/jbc.M802973200

[bib36] van de Vijver MJ, He YD, van't Veer LJ, Dai H, Hart AA, Voskuil DW, Schreiber GJ, Peterse JL, Roberts C, Marton MJ, Parrish M, Atsma D, Witteveen A, Glas A, Delahaye L, van der Velde T, Bartelink H, Rodenhuis S, Rutgers ET, Friend SH, Bernards R (2002) A gene-expression signature as a predictor of survival in breast cancer. N Engl J Med 347: 1999–2009 12490681 10.1056/NEJMoa021967

[bib37] van't Veer LJ, Dai H, van de Vijver MJ, He YD, Hart AA, Mao M, Peterse HL, van der Kooy K, Marton MJ, Witteveen AT, Schreiber GJ, Kerkhoven RM, Roberts C, Linsley PS, Bernards R, Friend SH (2002) Gene expression profiling predicts clinical outcome of breast cancer. Nature 415: 530–536 11823860 10.1038/415530a

[bib38] Vranka JA, Sakai LY, Bachinger HP (2004) Prolyl 3-hydroxylase 1, enzyme characterisation and identification of a novel family of enzymes. J Biol Chem 279: 23615–23621 15044469 10.1074/jbc.M312807200

[bib39] Wang Y, Klijn JG, Zhang Y, Sieuwerts AM, Look MP, Yang F, Talantov D, Timmermans M, Meijer-van Gelder ME, Yu J, Jatkoe T, Berns EM, Atkins D, Foekens JA (2005) Gene-expression profiles to predict distant metastasis of lymph-node-negative primary breast cancer. Lancet 365: 671–679 15721472 10.1016/S0140-6736(05)17947-1

[bib40] Wassenhove-McCarthy DJ, McCarthy KJ (1999) Molecular characterization of a novel basement membrane-associated proteoglycan, leprecan. J Biol Chem 274: 25004–25017 10455179 10.1074/jbc.274.35.25004

[bib41] Zhao H, Langerød A, Ji Y, Nowels KW, Nesland JM, Tibshirani R, Bukholm IK, Kåresen R, Botstein D, Børresen-Dale AL, Jeffrey SS (2004) Different gene expression patterns in invasive lobular and ductal carcinomas of the breast. Mol Biol Cell 15: 2523–2536 15034139 10.1091/mbc.E03-11-0786 PMC420079

